# Spatiotemporal Distribution Characteristics of Nutrients in the Drowned Tidal Inlet under the Influence of Tides: A Case Study of Zhanjiang Bay, China

**DOI:** 10.3390/ijerph18042089

**Published:** 2021-02-21

**Authors:** Shuangling Wang, Fengxia Zhou, Fajin Chen, Yafei Meng, Qingmei Zhu

**Affiliations:** 1 College of Ocean and Meteorology, Guangdong Ocean University, Zhanjiang 524088, China; wishling@126.com (S.W.); fxzhou@gdou.edu.cn (F.Z.); mengyafei199001@sina.com (Y.M.); zhuqingmei1987@163.com (Q.Z.); 2 Key Laboratory for Coastal Ocean Variation and Disaster Prediction, Guangdong Ocean University, Zhanjiang 524088, China

**Keywords:** drowned-valley tidal inlet, phosphorus, nitrogen, numerical model, Zhanjiang Bay

## Abstract

The tidal dynamics and the characteristics of pollutant migration in the drowned-valley tidal inlet, a typical unit of coastal tidal inlets, are strongly influenced by geomorphological features. Along with the development of society and the economy, the hydrodynamic and water quality environment of the tidal inlet is also becoming more disturbed by human activities, such as reclamation of the sea and the construction of large bridges. In this study, a typical drowned-valley tidal inlet, Zhanjiang Bay (ZJB), was selected for the establishment of a model via coupling of a tidal hydrodynamic model and water quality numerical model. This model can be used to simulate the migration and diffusion of pollutants in ZJB. The spatial and temporal variation processes of water quality factors of the bay under the influence of special geomorphic units was simulated at the tidal-inlet entrance, the flood/ebb tidal delta, and the tidal basin. The results show that ZJB has strong tidal currents that are significantly affected by the terrain. Under the influence of the terrain and tidal currents, the phosphorus and nitrogen concentration at the flood-tide and ebb-tide moments showed obvious temporal and spatial differences in the ebb-tide delta, tidal-inlet entrance, flood-tide delta, and tidal basin. In this study, we analyzed the response mechanism of the water quality environment to the drowned-valley tidal inlet, and this can provide theoretical guidance and a basis for decision-making toward protecting the ecology and water security of ZJB.

## 1. Introduction

Tidal inlets are an important type of coastal dynamic geomorphology occurring on tidal coasts. They are unified dynamic geomorphological systems that are interrelated and influenced by the ebb-tide delta, tidal-inlet entrance, flood-tide delta, tidal basin, and other factors. The system is sensitive to the response and feedback of environmental pollution [1,2]. With the continuous development of the social economy, the hydrodynamic water quality environment of tidal inlets is also increasingly being disturbed by human activities, including land construction around the sea, large-scale bridge construction, land source pollution emissions, and fishery farming [3,4,5]. How to slow down the problem of water pollution in coastal areas, especially tidal inlets, to ensure water ecological security is an urgent scientific problem to be solved [6,7,8,9]. Inshore geomorphological units and human activity interference are important factors affecting water quality [10,11,12]. Therefore, understanding the response mechanism of the sea area water quality environment to special geomorphological units and human activities is the premise and basis for solving the problem of inshore water pollution.

With regard to the tidal inlet, which is a special geomorphologic unit, the literature, both domestic and foreign, is mainly focused on the following aspects:

(1) The stability of a tidal inlet. Among the studies on the special geomorphologic units in the tidal areas near the shore, the stability of the tidal inlets has always been one of the hot spots and difficulties in research on the dynamic geomorphologic evolution of estuaries and coasts. By analyzing the tidal prism (P) and the cross-sectional area in a tidal-inlet (A) relationship of the tidal inlets, the sectional morphology of the tidal inlet was analyzed to judge the stability of the tidal inlets [2,13]. In addition, some scholars studied the stability of the tidal inlets from the hydrodynamic process of the tidal inlets by means of numerical models and, at the same time, pointed out the critical coefficients existing for the tidal inlets [14,15,16]. Numerous studies have also discussed the stability of the tidal inlets from the perspective of the coastal erosion and sediment movement of tidal inlets based on the differences among the tidal shapes in a tidal inlet [17,18,19,20].
(2) The tidal prism and water exchange. The amount of tidal prism directly affects the water exchange capacity as well as the rules of pollutant migration and diffusion in the bay. The duration of the water exchange in the tidal inlet is an important index for the vitality of a semi-closed bay. For the calculation of the static tidal prism capacity, a formula for calculating the linear tidal capacity is often used [2]. By virtue of the numerical model and the remote sensing data from the satellites, more scholars calculated the dynamic tidal prism capacity of a single-tide tidal inlet or a multi-tide tidal inlet [21]. With regard to water exchange, there are relatively more concepts available, such as the half-exchange time, persistence time, impact time, renewal time, and water age. At present, most of the calculations for the exchange capacity of water have been carried out through the numerical models for the tidal flow established based on studies using the two-dimensional convection–diffusion mathematical model [22,23].
(3) The tidal wave hydrodynamic characteristics of the tidal inlets. Study of the hydrodynamic processes of tidal inlets has provided a hydrodynamic field for assessing the stability, coastal erosion, material transport, and water quality environment of a tidal inlet [24,25,26,27]. The numerical simulation study of tidal hydrodynamics began in the 1950s. It gradually developed from a one-dimensional model to a two-dimensional model, and the numerical simulation and calculations were mainly carried out according to the law of water movement. In the 1970s, with the continuous exploration and extension of the research on the two-dimensional hydrodynamic models of the coastal waters, many scholars began to study the three-dimensional numerical simulation of inshore tides. With three-dimensional numerical simulation, it is possible to realize the dynamic simulation of the tides across the scales of space and time [15,28,29]. The research on the response of the water quality environment to near-shore tidal branch geomorphologic units is comparatively lacking.

**Figure 1:**
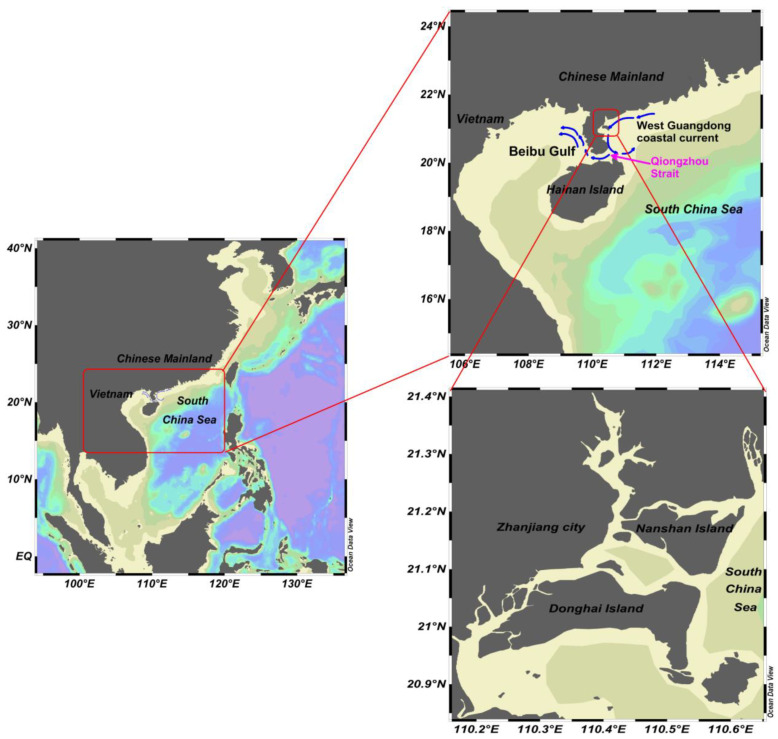
The location of Zhanjiang Bay (ZJB).

The western coast of Guangdong is located on the southwest side of the northern shelf of the South China Sea, which is connected with the Beibu Gulf by the Qiongzhou Strait. Zhanjiang Bay (ZJB) is a typical drowned-valley tidal inlet in this area, and the terrain is relatively flat. The special terrain makes the west Guangdong coastal current constitute the northern wing of the eastern cyclone vortex of the Qiongzhou Strait, and a part of the westward coastal current enters the Beibu Gulf westward through the Qiongzhou Strait (Figure 1). Studies have shown that the west Guangdong coastal current has a significant impact on the transport of pollutants and nutrients in the coastal waters west of the Pearl River Estuary, Qiongzhou Strait, and Beibu Gulf [30,31]. Therefore, this study selected ZJB, a typical drowned-valley tidal inlet in the west of Guangdong Province, to study the feedback effect of ecological environment of the bay on dynamic process in the northwest of the South China Sea, which can expand the understanding of the dynamic forcing mechanism of the coastal ecosystem in the northwestern South China Sea.

The MIKE3 Flow Model (FM) is a prominent three-dimensional water environment numerical simulation modeling, and MIKE Ecological Modeling (ECO Lab) is a piece of numerical simulation software for ecological modeling, both of which were developed by the Danish Hydraulic Institute (HDI), and these models have been applied worldwide [32,33,34,35]. MIKE3 FM is based on a flexible mesh approach, and it has been developed for applications within oceanographic, coastal, and estuarine environments. The MIKE3 FM hydrodynamic model and the MIKE ECO Lab model can be used to simulate the spatiotemporal distribution characteristics of nutrients in the drowned tidal channel under the influence of tides. This study provides a scientific basis for controlling the water environment of ZJB, a typical drowned-valley tidal inlet.

## 2. Study Area

**Figure 2:**
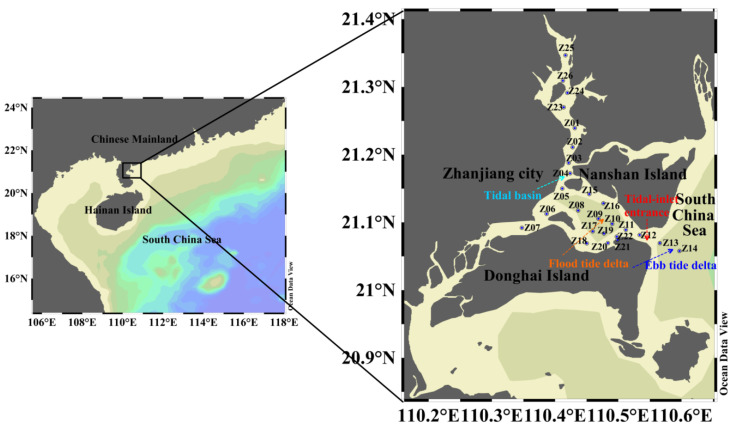
The study area showing sampling sites (Z1–Z26) in ZJB. ZJB can be roughly divided into four parts: the ebb-tide delta, tidal-inlet entrance, flood-tide delta, and tidal basin.

Zhanjiang Bay (ZJB), located at the northwestern coastal region of the South China Sea (Figure 2), is the largest drowned-valley tidal channel on the South China Coast. ZJB takes the shape of a tree branch, extending inland for approximately 50 km from south to north. It is surrounded by Nansan Island, Donghai Island, and Leizhou Peninsula. ZJB can be roughly divided into four parts according to its geomorphological features: the ebb-tide delta, tidal-inlet entrance, flood-tide delta, and tidal basin. ZJB has an expanse of water areas, with tides of large capability and strong force and stable beaches and water troughs, and represents the most dynamic, complex, and vulnerable marine environment in the South China Sea [36]. The drowning-valley-type tidal channel in ZJB has clear irregular semi-diurnal tidal features. The main tidal components M_2_, S_2_, O_1_, K_1_, M_4_, and MS_4_ are almost all introduced from the open sea. Due to the friction from the nearby islands and terrain, the characteristics of currents are more complicated.

In recent years, with the demands of ZJB’s economic development, the development of ports and channels, energy bases, harbor industries, and tidal flats in the bay have been included in the economic development plan of ZJB. In addition to the above projects, pollution discharge from land sources, fishery, and aquaculture in addition to port and shipping pollution have all changed the ZJB water environment to varying degrees, with the water quality affected to some extent [37]. Over recent decades, rapid economic development and urbanization have significantly impacted the ZJB environment. Eutrophication and harmful algal blooms have occurred frequently [38,39]. A scientific understanding of the response mechanism of the water quality environment of ZJB to special geomorphological units is the prerequisite and basis for solving the water pollution problem.

## 3. Methodology and Model Setup

### 3.1. Model Establishment

**Figure 3:**
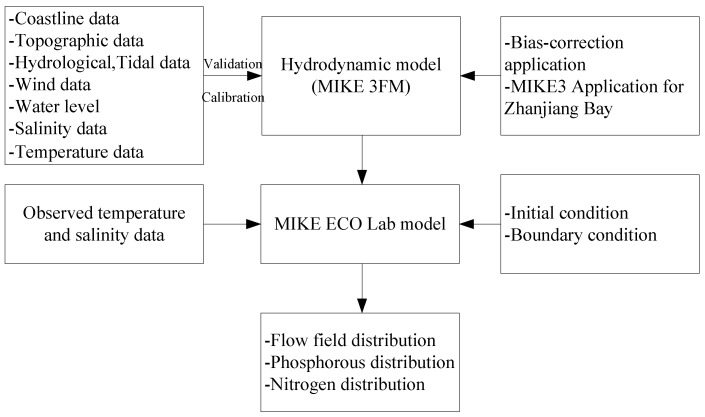
Schematic of the modeling framework for this study.

Based on the MIKE3 hydrodynamic module (Flow Model, FM) and water ecological module (MIKE ECO Lab) (MIKE Zero release 2014), we constructed a three-dimensional hydrodynamic model and nutrient diffusion model to simulate the ZJB hydrodynamic process and the migration and diffusion process of nutrients at the ebb-tide delta, tidal-inlet entrance, flood-tide delta, and tidal basin. Comparison and analysis of the distribution of nutrients at the flood tide and ebb tide can provide a scientific basis for the optimization of the pollution sources in ZJB. The research framework of this study is shown in Figure 3.

### 3.2. Difference Scheme and Discrete Equation

Considering the conditions for the definite solution, we can solve the equation using the discrete form. The MIKE3 model applies C-type grids in rectangular coordinates and adopts a stable alternating direction implicit (ADI) scheme for the discrete difference. The equation matrix applies double sweep for the solution, which has second-order accuracy.

Owing to a large area of tidal flats in the research area, which become dried out or flooded due to high or low tides, the existence of variable boundaries should be considered to accurately simulate the phenomenon. In our model, the dry–wet grid method was adopted to deal with flooding. When the water level drops to a certain point and the water depth is less than the critical value of the dry point, the point is omitted from the calculation. When the sea water rises and its depth is greater than the critical depth at the wet point, the calculation is re-added. If the value of the water depth at the dry and wet points is extremely large, the accuracy of the results is affected. If the value is extremely small, an unstable calculation may occur.

### 3.3. Boundary/Initial Conditions

**Figure 4:**
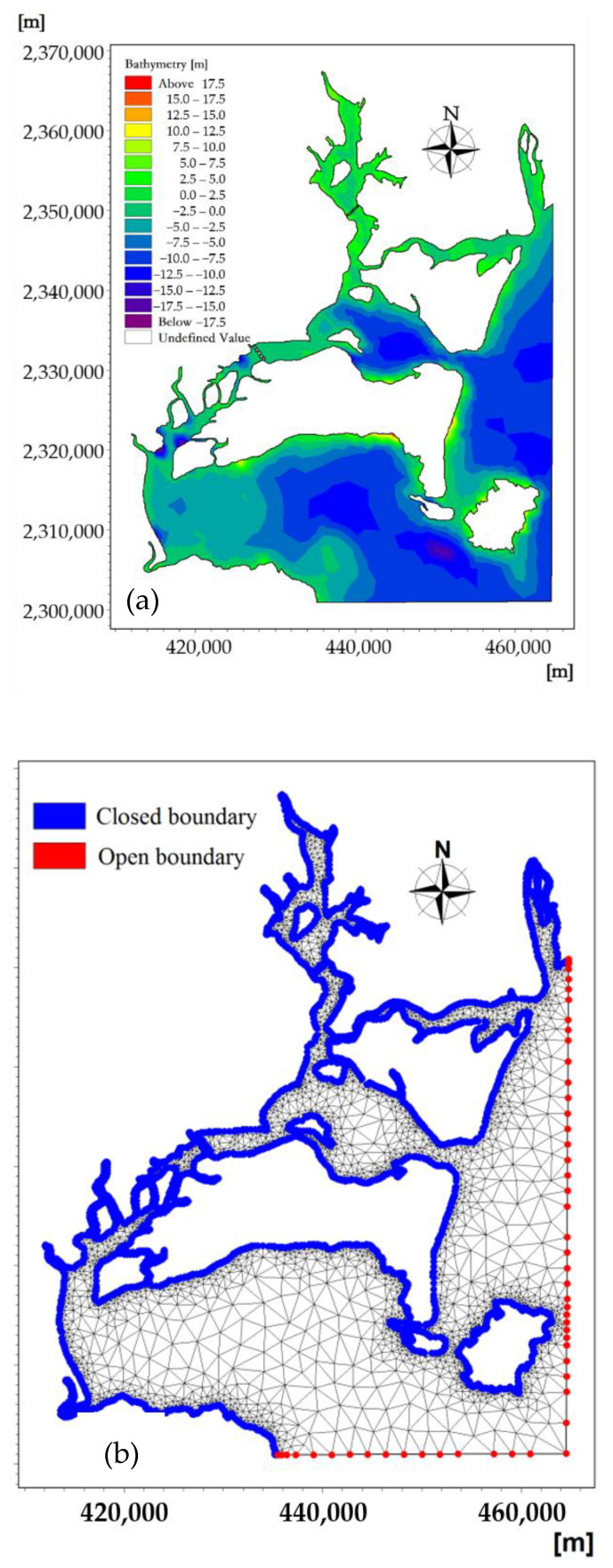
Bathymetry (**a**) and computational flexible grid mesh (**b**).

The boundary conditions and initial conditions of the model are as follows (Figure 4):

Closed boundary: Code1 in the model calculation is the closed boundary, that is, the land boundary, U = 0 or V = 0.

Open boundary: The south boundary and the east boundary are the open boundaries in the numerical model.

The model considers external forces, such as the tidal current, temperature, salinity, and wind. At the opening boundary, the water level data come from the Oregon State University (OSU) Tidal Inversion Software (OTIS). This study selected six main tidal components, M_2_, S_2_, O_1_, K_1_, M_4_, and MS_4_, as the external forces of the open boundary. The wind data were from the European Center for Medium-Range Weather Forecasts (ECMWF) with a high resolution of 6 h by 6 h. The east component U and north component V of the wind speed at 10 m height from the sea surface, with a spatial resolution of 0.125° × 0.125°, were interpolated to each grid and generated as a wind field forcing file.

The temperature and salt open boundary data came from National Centers for Environmental Prediction (NCEP) Global Ocean Data Assimilation System (GODAS) monthly average data, and the initial temperature and salinity field came from the monthly average data of the World Ocean Atlas 2013 V2 (WOA13). The above data were taken as the model-driven forcing conditions and initial conditions for the interpolation method. The temperature and salt boundary conditions of the water quality model were the same as those of the hydrodynamic model. The nutrient boundary conditions and background values were interpolated with the measured values. The model was used to determine the variation of the dry–wet inundation grid at the start of the open boundary, and the minimum water depth was set at 0.05 m.

### 3.4. Model Schematization

The detailed bathymetry in numerical modeling plays a critical role in achieving accurate hydrodynamic simulations, especially in the drowned-valley tidal inlet. The modeling area of the horizontal plane is 110.1396°–110.6305° E and 20.9174°–21.5236° N. The model coastline adopted the high-precision data issued by National Oceanic and Atmospheric Administration’s National Geophysical Data Center (NOAA/NGDC) and was adjusted using the sea chart data of the coastal sea area to obtain accurate coastline data. The terrain data in the coastal area were obtained from the ETOPO1 topographic data with resolution of 1′ × 1′′ and adjusted using the measured terrain data. The ETOPO1 is a 1 arc-minute global relief model of Earth’s surface that integrates land topography and ocean bathymetry.

The coastline and topographic data obtained by using the above two kinds of data can accurately reflect the actual situation of the calculation area. The simulation grid cell selection for the study area is a flexible mesh (FM) or unstructured mesh, where the triangular cells of bathymetry are used to optimize the simulation, with small sizes near land domains and larger sizes in offshore settings. The triangular element sizes are about 800–1000 m offshore, with a total triangular area of 13,636 elements and 8820 nodes. The bathymetry and computational flexible grid mesh are as shown in Figure 4.

To obtain the tidal current dynamic data and water quality concentration data of ZJB, a total of 26 observation stations were set up for regular voyages (Figure 2). The hydrometeorological parameters observed at each station mainly include the water depth, water temperature, salinity, flow velocity, flow direction, wind speed, and wind direction. Water samples were also collected at each site for the analysis of the chemical parameters. The samples were taken according to the standard level of oceanographic investigation and research. The analyzed water quality parameters mainly included dissolved oxygen and nutrients. Water samples for nutrient analysis were immediately filtered through acid cleaned 0.45 μm acetate cellulose filters. The filtrates were collected in pre-cleaned polyethylene bottles and stored at −20 °C until laboratory analysis. Nutrients were determined using the Skalar San^++^ continuous flow analyzer. The water level of continuous observation stations was automatically collected by an SBE-26 wave tide meter, while the water level of the conventional observation station was measured by conductivity-temperature-depth (CTD) sensors.

The dissolved inorganic nutrients included phosphate (PO_4_-P) and dissolved inorganic nitrogen (DIN). DIN is defined as the sum of the dissolved nitrate (NO_3_-N), nitrite (NO_2_-N), and ammonium (NH_4_-N). A total of four observation stations (Z4, Z8, Z11, and Z21) were set up for continuous voyages, and the continuous observation time was 75 h. Spring tides and neaps of different seasons were selected for observation. When conducting continuous observations, the measured hydrological parameters were the same as the above, and water samples were collected every three hours for chemical parameter analysis.

### 3.5. Model Calibration and Validation

The parameters of the module were set as follows: calculation of the time-step interval, 30 min; initial water level, 0 m; roughness height data, 0.1 m; and nitrate first-order decay rate at 20° C, 0.1/day. The computing time was from 14 August 2017, 0 points, to 24 points on the 16th. The model was validated based on the tide level and nutrition concentration. Measured data obtained from 14 to 16 August were used to validate the model. There were four continuous observation stations (Z4, Z8, Z11, and Z21) (Figure 2), and the water levels and nutrient concentrations monitored at these continuous observation stations can be used for model validation.

In this study, the numerical simulation is divided into two parts: hydrodynamic simulation and water quality simulation. The main calibration parameters for hydrodynamic simulation are Manning number and Courant–Friedrich–Levy (CFL), and the main calibration parameters for water quality simulation are pollutant diffusion coefficient and degradation coefficient.

The Manning number and Courant–Friedrich–Levy (CFL) were specified considering the variable depth for calibrating the MIKE 3 FM model. The CFL number is accounted in the MIKE 3 FM for the numerical stability criterion. The calibration process aims to match simulated results and observed data, including the water level and salinity concentration in different locations, by changing the Manning number and CFL in MIKE 3. The Manning number is defined as a function of water depth and can be calculated based on depth and drag coefficient [40]. The CFL number is accounted in the MIKE 3 FM module for the numerical stability criterion. In order to ensure the accuracy of the model, according to the results of model verification, the Manning number and CFL number in the model are 0.018 s/m^1/3^ and 0.8, respectively.

Due to the high requirement of global sensitivity analysis for computer and the long running time of the model, this study carried on the local sensitivity, used the disturbance method, only changed one input parameter at a time, with the other parameters remaining unchanged to obtain parameter sensitivity of the law, and the water quality model parameters were continuously adjusted and calibrated [41,42]. Sensitivity analysis parameters include the diffusion coefficient and degradation coefficient of PO_4_-P and DIN. The results show that diffusion coefficient can be regarded as an insensitive parameter, while degradation coefficient has high sensitivity, and its sensitivity is that the degradation coefficient of PO_4_-P is greater than the degradation coefficient of DIN. On the basis of the hydrodynamic model, according to the results of local sensitivity analysis, the degradation coefficients of PO4-P and DIN are constantly adjusted according to the simulation conditions until the best calibration result is achieved. The diffusion coefficient of PO_4_-P and DIN is 1.6 m^2^/s, the degradation coefficient of PO_4_-P and DIN are 1.68 × 10^−7^/s and 5.1 × 10^−^^8^/s, respectively.

**Figure 5:**
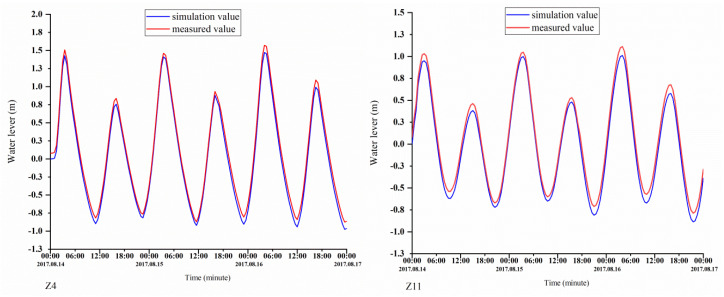

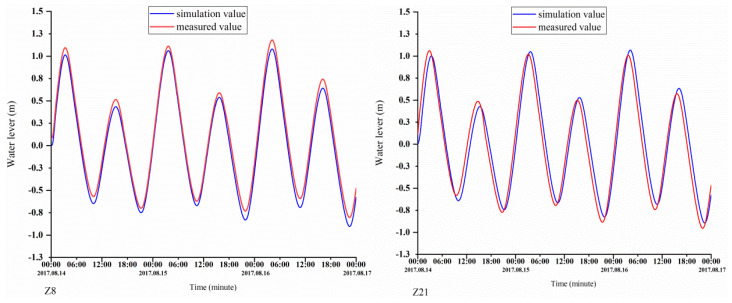
Verification of the tidal levels at the Z4, Z8, Z11, and Z21 stations.

**Figure 6:**
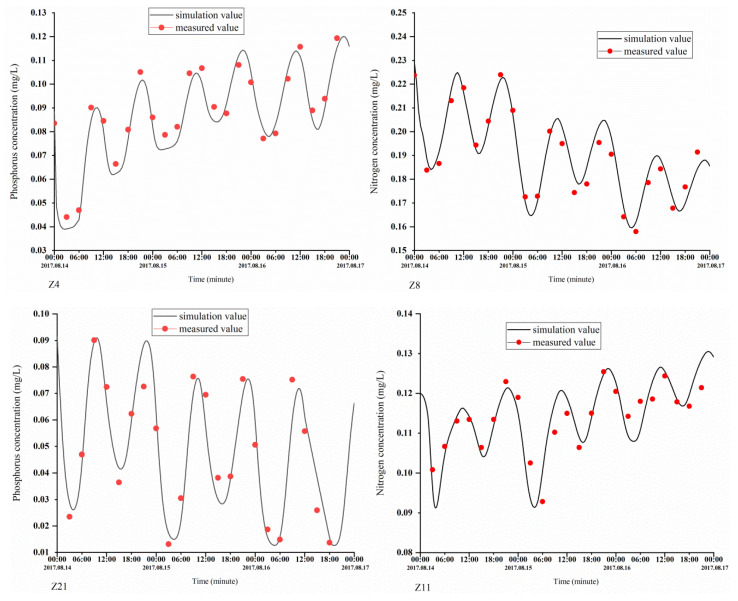
Validation of the phosphorus concentrations at the Z4 and Z21 stations and nitrogen concentrations at the Z8 and Z11 stations.

The simulation results were compared with the measurements. Figure 5 and Figure 6 show the validation results of the water level (Z4, Z8, Z11, and Z21), phosphorus concentrations (Z4 and Z21), and nitrogen concentrations (Z8 and Z11). The validation results demonstrate that the errors between the calculated and measured water levels were predominantly within 10 cm; in addition, the variation pattern of the calculated tidal current velocity was consistent with that of the measured tidal current velocity, and the errors between the calculated and measured nitrogen concentrations were within 20%. The calibration results demonstrate that the model produces relatively good simulation results, the model parameter selection was reasonable, and the calculation results can characterize the ocean currents in the study area.

## 4. Results and Discussion

### 4.1. Results of the Calculation of the Flow Field in ZJB

ZJB takes the shape of a tree branch and is the largest drowned-valley tidal channel on the South China Coast. Under the influence of the terrain, the flow fields of the ebb-tide delta, tidal-inlet entrance, flood-tide delta, and tidal basin are clearly different. The width of each segment narrows going upstream such that the energy is concentrated after the tidal wave enters, and the upstream tidal range increases, thereby increasing the tidal capacity. In the narrowed section, the scouring force of the tide is strengthened, and the depth of the deep groove is stabilized.

Due to the influence of the terrain, the tide in ZJB becomes more complicated when it rises and falls. In the throat section of the mouth, due to the narrow tube effect, the flow velocity is particularly high at the ebb and flood tide. The drowning-valley-type tidal channel in ZJB has obvious irregular semi-diurnal tidal features. The main tidal components M_2_, S_2_, O_1_, K_1_, M_4_, and MS_4_ are almost all introduced from the open sea. There is no river influence in and out of ZJB, and the flow power is mainly the tidal current [43]. Due to the friction from nearby islands and terrain, the current characteristics are more complicated [44].

Based on the calculated results, the ZJB area has strong tidal currents that are significantly affected by the terrain. ZJB has an irregular semi-diurnal tidal pattern, with two high tides and two low tides in one day, with diurnal inequality. Influenced by Donghai Island, Nanshan Island, and the continental shoreline, a stable tidal flood and ebb-tide channel and deep trough have been formed [43]. In the tidal-inlet entrance and bay, affected by the terrain, the tidal current is reciprocating [43]. The flood tide is mainly westward WSW–WNW, the ebb tide is mainly in the ESE direction, and the ebb-tide speed is generally greater than the flood-tide speed. Affected by the terrain, the tidal level gradually increases from the outside of the bay to the inside of the bay, and the tidal range increases from the outside of the bay to the inside of the bay.

**Figure 7:**
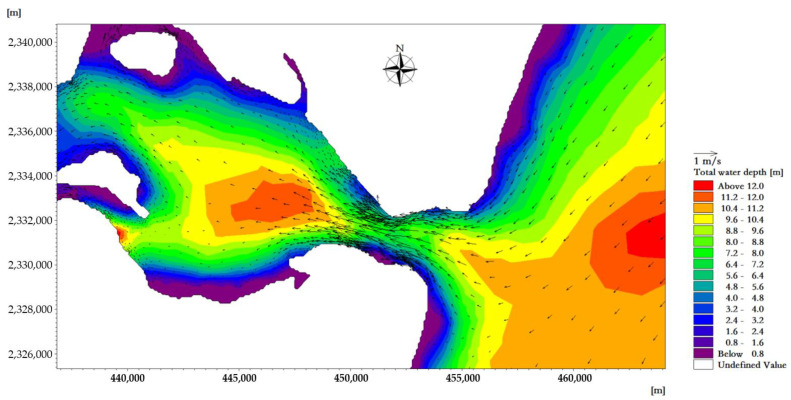
Flow field plane distribution near the tidal inlet at the moment when the velocity of the flood tide reaches its maximum.

**Figure 8:**
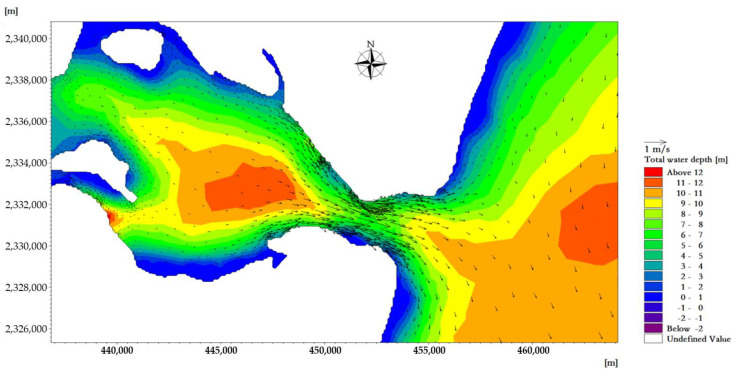
Flow field plane distribution near the tidal inlet at the moment when the velocity of the ebb tide reaches its maximum.

The average high water level of the tidal-inlet entrance is 0.97 m, and the low water level is 0.80 m. The reciprocating flow is in the direction of the channel. The flow rate of the ebb tide is greater than that of the flood tide. The velocity is strongest near the tidal inlet of the bay. At the moment when the velocity of the flood tidal current of a tide reaches its maximum, the tidal current direction in the deep-water area runs from east to west and is mainly perpendicular to the sea contour line. The maximum velocity at the tidal outlet was about 0.98 m/s (Figure 7). At the moment when the velocity of the ebb tidal current of a tide reaches its maximum, the tidal current direction in the deep-water area is from west to east, and it is mainly perpendicular to the sea contour. The maximum velocity at the tidal outlet was about 1.05 m/s (Figure 8).

**Figure 9:**
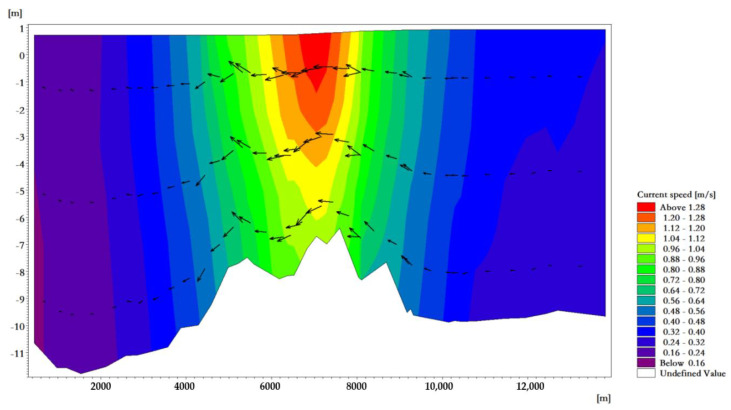
Flow field vertical distribution near the tidal inlet at the moment when the velocity of the flood tide reaches its maximum.

**Figure 10:**
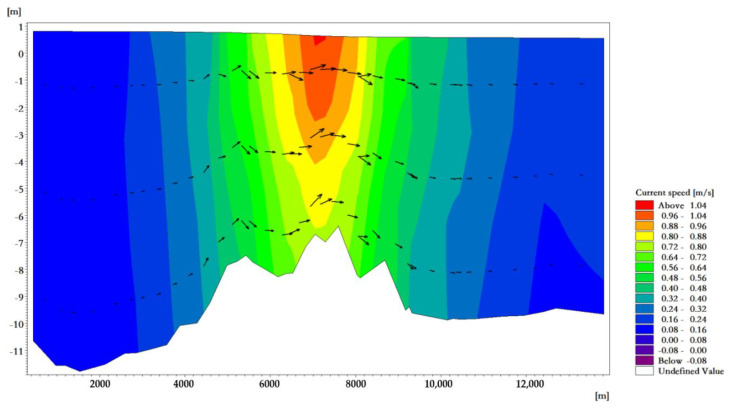
Flow field vertical distribution near the tidal inlet at the moment when the velocity of the ebb tide reaches its maximum.

The maximum surface velocity is distributed in the tidal-inlet entrance, and the maximum flow velocity on the surface of the flood and ebb tide was 0.98 and 1.05 m/s, respectively. In the vertical distribution of velocity, the maximum velocity appears in the surface layer and in the middle layer; however, the difference of velocity between the surface layer, the middle layer, and the bottom layer was not large (Figure 9 and Figure 10). It can be seen from the results that flood-/ebb-tide delta and tidal-inlet entrance water bodies had a stronger exchange with the outer sea, while the tidal basin water bodies had a weaker exchange with the outer sea bodies, which is similar to other research results [45].

**Figure 11:**
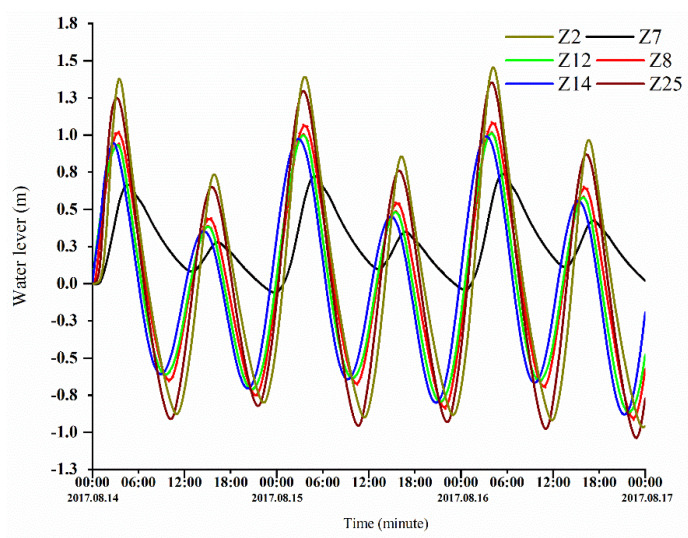
The sea level time series during the three days at different stations in ZJB.

The three-day time series of sea level variations at different stations in ZJB during the tide gauge-mooring period from 8 to 10 July 2017 is shown in Figure 11. The water level at the tidal inlet changes the most, followed by the flood tidal delta and the ebb tidal delta.

### 4.2. Results of the Spatiotemporal Distribution of the Phosphorus Concentration

**Figure 12:**
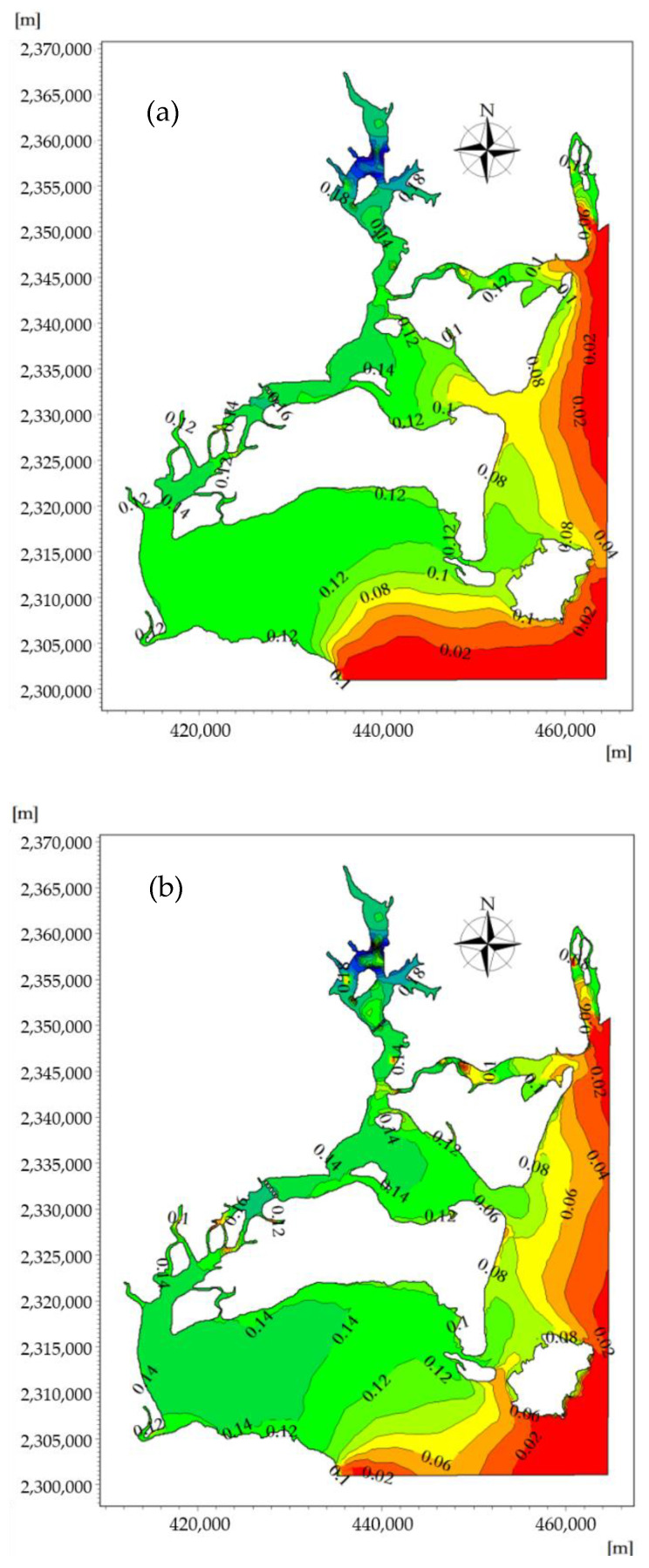
The spatiotemporal distribution of phosphorus concentrations across the modeling domain at the moment when the velocity of the flood tide (**a**) and ebb tide (**b**) reaches its maximum (mg/L).

Based on the MIKE3 hydrodynamic module (Flow Model, FM) and considering the influence of temperature and salt in ZJB, the two-dimensional and vertical three-dimensional hydrodynamic processes of ZJB were calculated. Combined with the measured nutrient concentration in ZJB, the MIKE ECO Lab model was used to simulate the distribution of nutrients in ZJB during the tidal cycle. The spatiotemporal distribution of the phosphorus concentrations at the flood tidal delta, tidal-inlet entrance, the ebb tidal delta, and the tidal basin during flood tide and ebb tide are shown are shown in Figure 12a,b, respectively.

To quantify the spatial variations of the nutrient concentration across the modeling domain, the maximum and minimum phosphorus concentrations during the flood tidal and ebb tidal were calculated. The results showed that the spatiotemporal distribution of phosphorus in ZJB was significantly different over the flood-tide duration and ebb-tide duration. The phosphorus concentration field in the offshore areas changed periodically with the movement of the tidal current.

**Figure 13:**
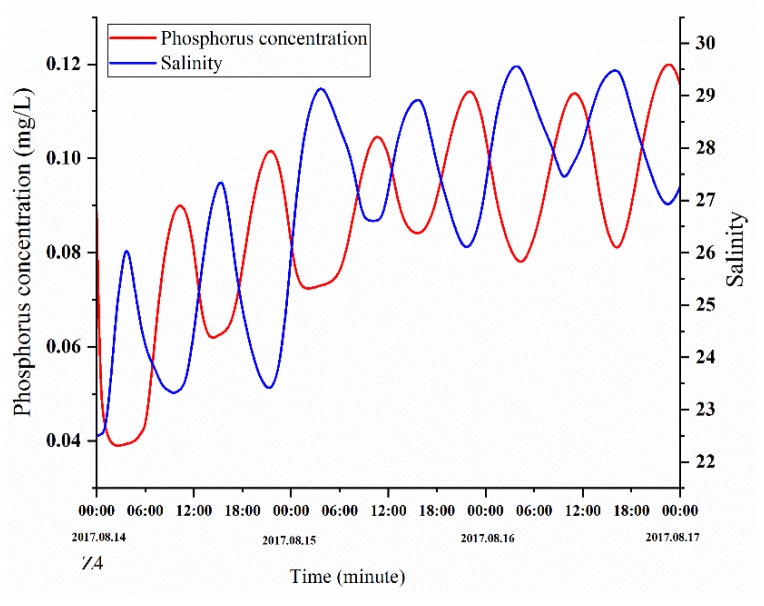
Temporal distribution of phosphorus concentrations and salinity at station 4 during the three days.

Areas with high concentrations of pollutants were primarily concentrated near the discharge outlet (urban areas and aquaculture areas), and the contour lines of the phosphorus concentration distribution were relatively dense. The concentration of phosphorus in the tidal basin of ZJB was the highest, and the concentration of phosphorus in the tidal-inlet entrance was the lowest at both the flood-tide and ebb-tide moments. The variation characteristics of the phosphorus concentration were roughly consistent with the depth contour. Station 4 in Figure 12 is within the tidal basin of ZJB, where the velocity is relatively slow, and fish farming (oysters, fish, shrimp, etc.) and urban sewage are concentrated. In estuaries and near-shore areas, due to the influence of the tidal cycle and land-source pollution, the distribution of phosphorus concentration has a negative correlation with salinity [36]. Under the influence of tidal cycles and land-source pollution, the concentration phosphorus in station 4 shows a fluctuation in levels (Figure 13).

During the slack water period, the range of phosphorus concentrations at the ebb-tide delta, tidal-inlet entrance, flood-tide delta, and tidal basin were, respectively, 0.025–0.071, 0.071–0.083, 0.083–0.142, and 0.142–0.280 mg/L. During the ebb slack period, the phosphorus concentration ranges at ebb-tide delta, tidal-inlet entrance, flood-tide delta, and tidal basin were, respectively, 0.045–0.083, 0.083–0.129, 0.129–0.155, and 0.155–0.280 mg/L. Through the effect of the ebb tide, some pollutants can also be brought to the outer bay area to be purified in the outer sea, reducing the possibility of water pollution in the bay.

However, the water environment is poor due to the small area, narrow topography, small flow velocity of part of the tidal basin, and because the coastal area of this part is the location of a densely populated area that includes the industrial zone and port of Zhanjiang City, whereby the main terrestrial pollutants are also discharged into the sea area here [38,39]. The tidal inlets connecting oceans to estuaries profoundly influence the ecology of estuarine water bodies [4]. Studies have analyzed the dissolved oxygen dynamics [46], nutrient dynamics, phytoplankton communities [47,48], and composition of fish assemblages [4] at coastal tidal inlets. Chinese white dolphin, mangrove crabs, and sea stars are common in ZJB, some studies have shown that the spatiotemporal distribution characteristics of nutrients and tides in ZJB and coastal urbanization can affect the ecology of these species [49,50,51].

**Figure 14:**
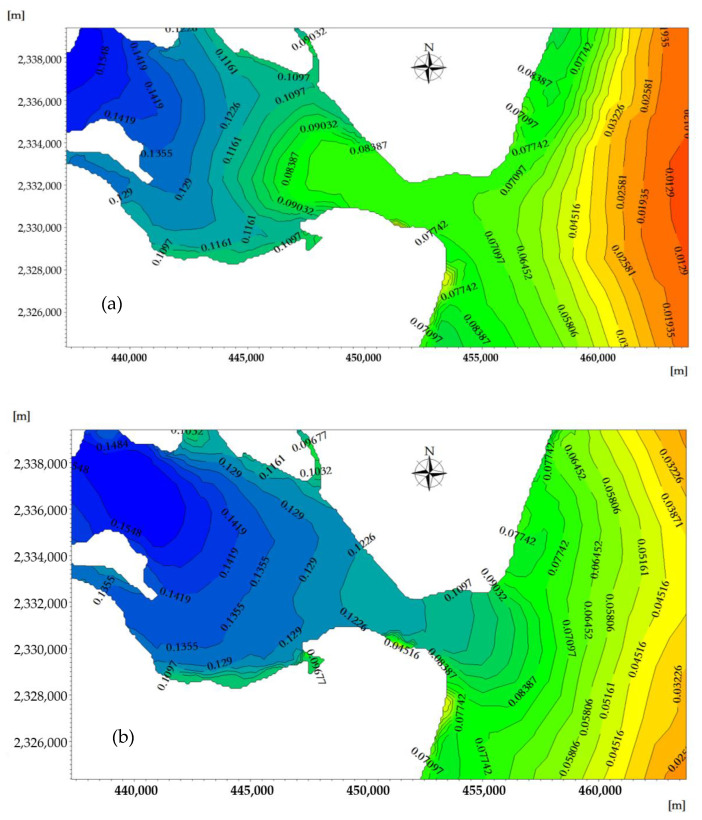
The spatiotemporal distribution of the phosphorus concentration near the tidal inlet at the moment when the velocity of the flood tide (**a**) and ebb tide (**b**) reaches its maximum (mg/L).

At the moment when a spring tide reaches high tide, the flood tidal current is about to turn into an ebb tidal current, and the phosphorus-affected area reaches its maximum during the rise of the flood tide. Due to the effect of the westward flood tidal current, the phosphorus concentration distribution migrates westward along the tidal-inlet entrance, and the seawater with low phosphorus content in the open sea enters ZJB through the tidal-inlet entrance. The concentration line of phosphorus curves from the ebb-tide delta to the flood-tide delta, and the 0.06–0.08 mg/L concentration envelope line penetrates into the flood-tide delta from the tidal-inlet entrance. The sea areas where the phosphorus concentration is 0.06–0.08 mg/L have an area of 63.11 km^2^, and the sea areas where the phosphorus concentration is in the range of 0.08–0.14 mg/L have an area of 65.67 km^2^ (Figure 14a).

At the moment when a spring tide reaches low tide, the ebb tidal current is about to turn into a flood-tide current, and the phosphorus-affected area reaches its maximum during the rise of the ebb tide. Due to the effect of the eastward flood tidal current, the phosphorus concentration distribution migrates eastward along the tidal-inlet entrance, and the seawater with higher phosphorus concentration in ZJB enters the South China Sea through tidal-inlet entrance.

The concentration line of phosphorus curves from the flood-tide delta to the ebb-tide delta, and the 0.06–0.08 mg/L concentration envelope line penetrates into the ebb-tide delta from tidal-inlet entrance. The sea areas where the phosphorus concentration is greater than 0.06–0.08 mg/L have an area of 63.98 km^2^, and the sea areas where the phosphorus concentration is in the range of 0.08–0.14 mg/L have an area of 88.55 km^2^ (Figure 14b). Under the influence of the terrain and tidal current, the phosphorus concentration throughout the duration of the flood tide and ebb tide shows clear temporal and spatial differences in the ebb-tide delta, tidal-inlet entrance, flood-tide delta, and tidal basin [52].

### 4.3. Results of Spatiotemporal Distribution of Nitrate Concentration

**Figure 15:**
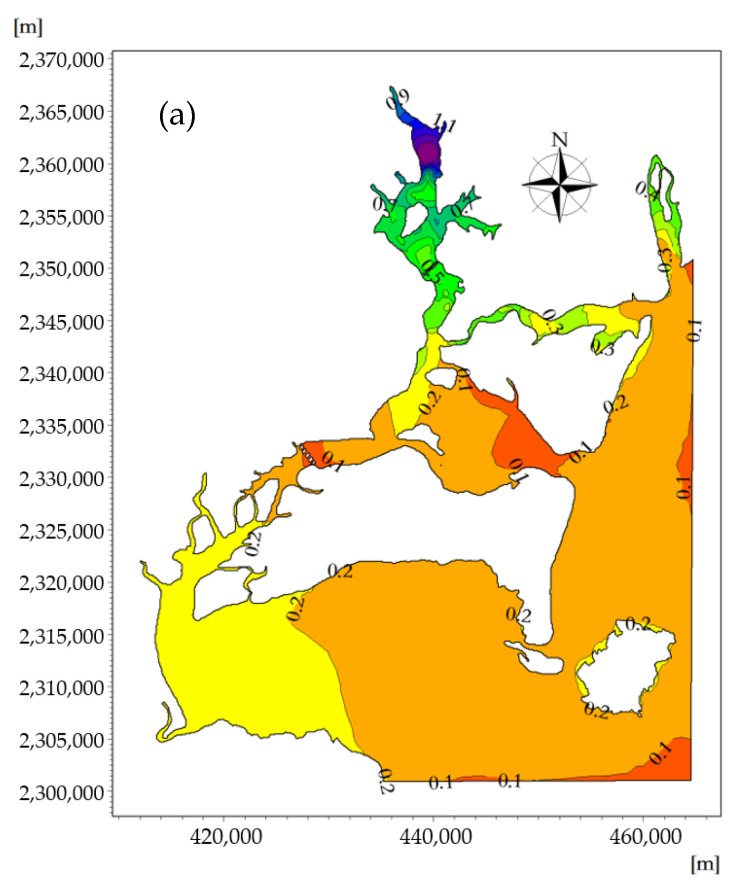

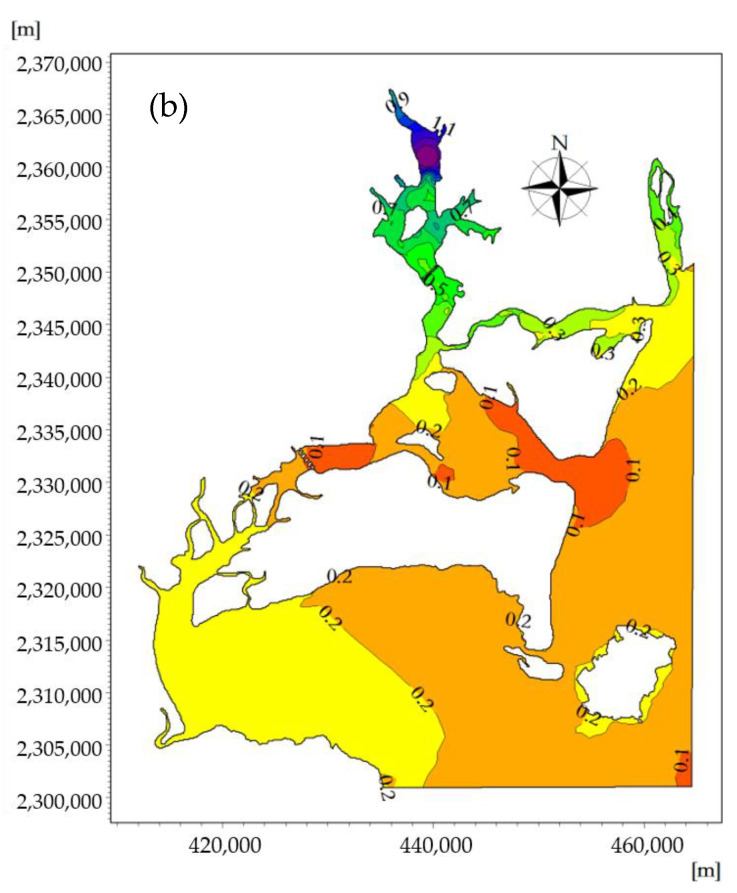
The spatiotemporal distribution of the nitrate concentration across the modeling domain at the moment when the velocity of the flood tide (**a**) and ebb tide (**b**) reaches its maximum (mg/L).

The spatiotemporal distribution of the nitrogen concentrations at the flood tidal delta, tidal-inlet entrance, ebb tidal delta, and tidal basin during the flood and ebb tides are shown in Figure 15a,b, respectively. The spatial distribution of the nitrogen concentration is similar to that of phosphorus concentration due to the effects of the land-source sewage discharge and aquaculture, and the concentration of the tidal basin is the highest during the period of the flood and ebb tides. In the throat section of the tidal-inlet entrance, due to the narrow tube effect, the velocity of the flow is particularly large at the time of flood and ebb tides, the turbulent diffusion is strong, and the concentration of nitrogen at the tidal-inlet entrance is the lowest.

With the exception of the tidal basin, the nitrogen concentration in other waters of ZJB was within the second-class range of water quality standards, and the nitrogen concentration was relatively low. The spatial and temporal distribution of phosphorus was significant at the time of flood and ebb tides, while the spatial and temporal difference of nitrogen was relatively small, especially in the flood/ebb tidal delta. During the flood- and ebb-tide periods, the nitrogen concentration in the flood/ebb tidal delta was mostly within the range of 0.1–0.2 mg/L. During the slack water period, the nitrogen concentration ranges at the ebb-tide delta, tidal-inlet entrance, flood-tide delta, and tidal basin were, respectively, 0.098–0.127, 0.097–0.106, 0.072–0.174, and 0.174–1.405 mg/L. During the ebb slack period, the nitrogen concentration range at the ebb-tide delta, tidal-inlet entrance, flood-tide delta, and tidal basin were, respectively, 0.135–0.058, 0.058–0.098, 0.098–0.193, and 0.193–1.412 mg/L.

**Figure 16:**
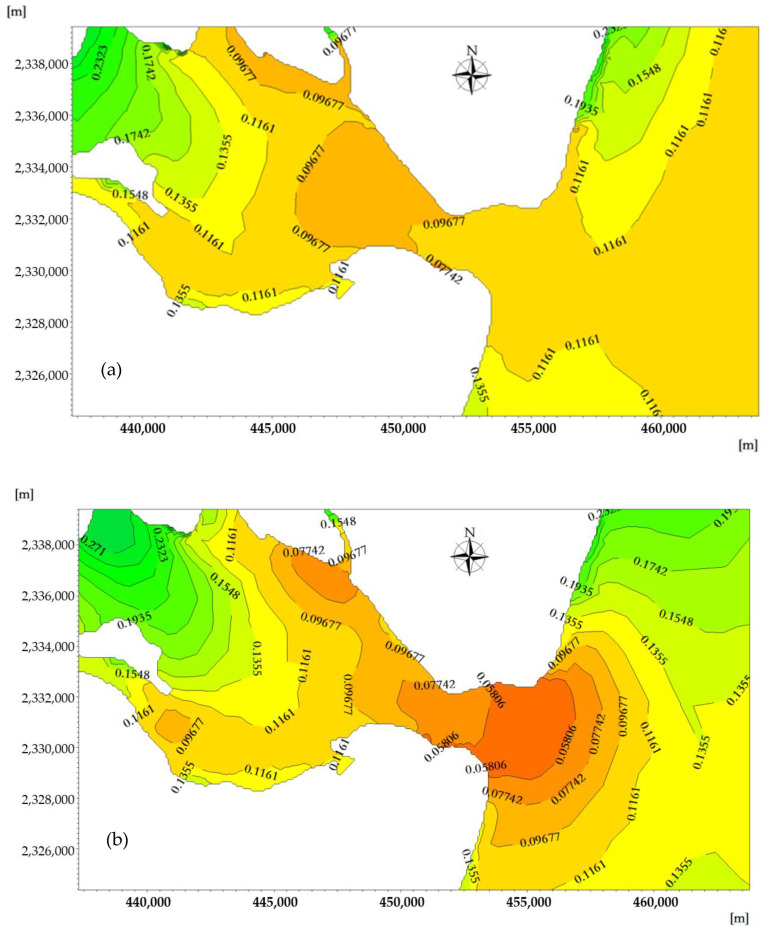
The spatiotemporal distribution of the nitrate concentration near the tidal inlet at the moment when the velocity of the flood tide (**a**) and ebb tide (**b**) reaches its maximum (mg/L).

At the moment when a spring tide reaches high tide, the flood tidal current is about to turn into an ebb tidal current, and the nitrogen-affected area reaches its maximum during the rise of the flood tide. Due to the effect of the westward flood tidal current, the nitrogen concentration distribution migrates westward along the tidal-inlet entrance, and the seawater with a low nitrogen content in the open sea enters ZJB through the tidal-inlet entrance. The concentration line of nitrogen curves from the ebb-tide delta to flood-tide delta, and the 0–0.1 mg/L concentration envelope line penetrates into the flood-tide delta from the tidal-inlet entrance. The sea areas where the nitrogen concentration is in the range of 0–0.1 mg/L have an area of 27.74 km^2^ (Figure 16a).

At the moment when a spring tide reaches low tide, the ebb tidal current is about to turn into a flood-tide current, and the nitrogen-affected area reaches its maximum during the rise of the ebb tide. Due to the effect of the east-westward flood tidal current, the nitrogen concentration distribution migrates eastward along the tidal-inlet entrance, and the seawater with a higher nitrogen concentration in ZJB enters the South China Sea through the tidal-inlet entrance. The concentration line of nitrogen curves from the flood-tide delta to the ebb-tide delta, and the 0–0.1 mg/L concentration envelope line penetrates into the ebb-tide delta from the tidal-inlet entrance. The sea areas where the nitrogen concentration is in the range of 0–0.1 mg/L have an area of 57.16 km^2^ (Figure 16b) [53].

The nitrogen concentration field in the offshore areas changes periodically with the movement of the tidal current. Under the influence of the terrain and tidal current, the nitrogen concentrations at the flood-tide and ebb-tide moments show obvious temporal and spatial differences at the ebb-tide delta, tidal-inlet entrance, flood-tide delta, and tidal basin. Decreased water velocities in the tidal basin generally lead to the accumulation of nutrients. Nutrient concentrations are also expected to be higher due to the reduced exchange with the more oligotrophic ocean water [4].

## 5. Conclusions

Based on the analysis of natural conditions of the ZJB areas, the spatial and temporal variation processes of water quality factors of the bay under the influence of special geomorphic units were simulated at the tidal-inlet entrance, the flood/ebb tidal delta, and the tidal basin using the MIKE3 FM hydrodynamic model and the MIKE ECO Lab model. The results of the present study can serve as a reference for the numerical simulation of similar projects. The main conclusions of the present study are as follows.

(1) ZJB has strong tidal currents that are significantly affected by the terrain. In the narrowed section, the scouring force of the tide is strengthened, and the depth of the deep groove is stabilized. Due to the influence of the terrain, the tide in ZJB becomes more complicated when it rises and falls. In the throat section of the mouth, due to the narrow tube effect, the flow velocity is particularly high during ebb and flood tides. There are significant differences in the tidal current velocity between the deep-water areas and the shoals.
(2) Under the influence of the terrain, the nutrient concentration changes greatly at the tidal-inlet entrance, flood/ebb tidal delta, and tidal basin with the change of the tide. The nutrients migrate southwestward with the flood tidal current and northeastward with the ebb tidal current. The dilution and dispersion of the nutrients are affected by the ocean currents in different tidal periods. At the time of flood tide and ebb tide, affected by the velocity and water level, the concentration of phosphorus in the tidal basin demonstrated a slight change while changing greatly at the ebb-tide delta, tidal-inlet entrance, and flood-tide delta. Except for the tidal basin, the nitrogen concentration in other waters of ZJB was within the second-class range of the water quality standard, and the nitrogen concentration was relatively low. Under the influence of the terrain and tidal current, the phosphorus concentration at the flood-tide and ebb-tide moments showed clear temporal and spatial differences at the ebb-tide delta, tidal-inlet entrance, flood-tide delta, and tidal basin.

## Data Availability

The data presented in this study are available on request from the corresponding author.
